# 

**Published:** 1889-08

**Authors:** 


### THE SOUTHERN DENTAL ASSOCIATION.

The Southern Dental Association will convene in Galveston, Texas, on Tuesday morning, August 20th, 1889. The committee of arrangements are sparing no pains or expense to make it a success. It is an opportunity not to be lost, and no dentist can afford to miss it. Here he will witness operations and receive instructions from the most skillful and earnest members of the profession, and he will carry home new thoughts and ideas and such pleasant recollections of his visit that he will want to come again. We want not only every dentist, but his family. This will be a rare opportunity for recreation and pleasure. Galveston, noted for her hospitality, will not be behind on this occasion. The hotels have offered greatly reduced rates, and we have assurance of the same rate as given by the railroads for the semi-centennial, due notice of which will be given through the press.

Fraternally yours,

W. O. CARRUTHERS,

Chairman Committee Arrangements.

### To Readers and Correspondents.

Communications intended for publication, and exchange journals and papers, should be addressed to the Editor of The American Journal of Dental Science, No. 845 N. Eutaw St. Baltimore, Md. All letters relating to the business of the Journal, or containing cash remittances, to be directed to Messrs. Snowden & Cowman, No. 9 W. Fayette Street, Baltimore, Md. Articles for publication should be received by the Editor at least two weeks previous to the first of the month on which the number in which it is designed for them to appear, is issued.

☞ The American Journal of Dental Science will contain fifty pages of reading matter in each number, making a volume of about Six Hundred pages,

EXCLUSIVE OF ADVERTISEMENTS.

